# Supplementary material for: VEGF‐B signaling impairs endothelial glucose transcytosis by decreasing membrane cholesterol content
Source: EMBO Rep. 2020 May 24;21(7):e49343. doi: 10.15252/embr.201949343 (PMC7332976; doi:10.15252/embr.201949343)
Supplement: Supplementary file 1 — Appendix [file EMBR-21-e49343-s001.pdf]

APPENDIX

TABLE OF CONTENTS

Appendix Supplementary Materials	pp. 2-3
Appendix Table S1	p. 4
Appendix Table S2	p. 5
Appendix Table S3	pp. 6-8
Appendix Figure S1	pp. 9-10
Appendix Figure S2	pp. 11-12
Appendix Figure S3	pp. 13-14
Appendix Figure S4	pp. 15-16
Appendix Figure S5	pp. 17-18
Appendix Figure S6	pp. 19-20

SUPPLEMENTARY MATERIALS

Cells:

Primary human umbilical vein endothelial cells (HUVEC), primary human brain microvascular endothelial cells (HBMEC) and primary human heart microvascular endothelial cells (HCMEC) were from PromoCell (Heidelberg, Germany). Mouse pancreatic endothelial cells (MS1) were purchased from ATCC.

Cell culture media:

Endothelial Cell Growth Medium 2 (for HUVEC and HBMEC), Endothelial Cell Growth Medium MV2 (for HCMEC and MS1) and corresponding basal medias (w/o SupplementMix) were from PromoCell, (Heidelberg, Germany). Dulbecco´s Modified Eagle Medium, Opti-MEM with GlutaMAX, heat-inactivated fetal bovine serum (FBS) and phosphate buffered saline (PBS) were from Gibco, Life Technologies.

Proteins:

Recombinant mouse vascular endothelial growth factor B_{167} (VEGF-B_{167}) derived from *E. coli* and recombinant mouse vascular endothelial growth factor B_{186} (VEGF-B_{186}) derived from Sf21 cells were from R&D Systems (Minneapolis, USA). Recombinant human vascular endothelial growth factor A_{165} (VEGF-A_{165}) derived from Sf21 cells were from R&D Systems (Minneapolis, USA).

Chemicals:

2-(N-(7-nitrobenz-2-oxa-1,3-diazol-4-yl)amino)-2-deoxyglucose (2-NBD-glucose) and 4,4-difluoro-5-methyl-4-bora-3a,4a-diaza-s-indacene-3-dodecanoic acid (C1-Bodipy 500/510 C12) (Bodipy-C_{12}-FA), 4',6-diamidino-2-phenylindole (DAPI) and ProlongGold mounting medium were from Invitrogen, Life Technologies. Growth factor reduced Matrigel was from BD Bioscience. Simvastatin, Filipin Complex from *Streptomyces filipinensis*, Phorbol 12-myristate 13-acetate (PMA), Methyl-β-cyclodextrin (MbCD) and water-soluble cholesterol were from Sigma-Aldrich. Native human low density lipoprotein:DiI (DiI-LDL) was from AbD Serotec. Kappa SYBR Fast qPCR kit was from Kappa Biosystems. EZ-Link Sulfo-NHS-Biotin and NEG-50 cryo-embedding medium were from Thermo Fisher Scientific. Complete protease inhibitor cocktail tablets and phosphatase inhibitor tablets PhosSTOP were from Roche Diagnostics GmbH. Steptavidin-sepharose high performance was from GE HealthCare. TopFluor® Cholesterol (23-(dipyrrometheneboron difluoride)-24-norcholesterol) was from Avanti Polar Lipids, Inc.

siRNAs:

Human siRNAs were purchased from Santa Cruz with the following order numbers: Control siRNA-A, sc-37007; *FLT1* siRNA, sc-29319; *NRP1* siRNA, sc-36038, *SLC2A1* siRNA, sc-35493; *KDR* siRNA, sc-29318; *LDLR* siRNA, sc-35802 and *SCARB1* siRNA, sc-44752.

Antibodies:

Antibodies are listed in **Table S1.**

Primers:

Primers were from Invitrogen and sequences are listed in **Table S2**.

Probes:

Selected genes from HBMEC microarray listed in **Table S3**.

Table S1. Antibodies

Target	Target Species	Source	Purchased from	Attachment
β-Actin	human, mouse	rabbit	Cell Signaling	
β-Actin	human, mouse	mouse	Abcam	
Caveolin 1	human	mouse	Abcam	
Calnexin (C20)	human, mouse	goat	Santa Cruz	
CD31	mouse, rat	goat	R&D Systems	
Connexin 43	human	rabbit	Zymed	
EEA1	human	rabbit	Novus Biological	
EEA1	human	mouse	Abcam	
Flotillin	human	rabbit	Sigma-Aldrich	
GAPDH	human	mouse	Novus Biological	
GLUT1	human, mouse	rabbit	Millipore	
GM130	human	mouse	BD Bioscience	
LAMP1	human	rabbit	Abcam	
LDLR	human	rabbit	Fitzgerald	
LDLR	human	mouse	Santa Cruz	
LIMPII/lpg85	human	rabbit	Novus Biological	
NRP1	human	goat	Santa Cruz	
Podocalyxin	mouse	goat	R&D Systems	
VEGFR1	human	rabbit	Novus Biological	
VEGFR1	mouse	goat	R&D Systems	
IgG	mouse	sheep	GE Healthcare	HRP
IgG	rabbit	donkey	GE Healthcare	HRP
IgG	goat	donkey	Santa Cruz	HRP
IgG	mouse	donkey	Invitrogen	Alexa 555
IgG	rabbit	donkey	Invitrogen	Alexa 488
IgG	goat	donkey	Invitrogen	Alexa 488
IgG	rabbit	donkey	Invitrogen	Biotin
IgG	goat	donkey	Invitrogen	Biotin

Table S2. Primer Sequences

Target	Species	Forward	Reverse
CD36	human	AACGGCTGCAGGTCAACCTA	ACATCACCACACCAACACTGA
CAV1	human	CAGAACAAACCTTTGGCGGG	GATGTCCCTCCGAGTCTACG
FLT1	human	ATTGTGGCCGAGCTGTTC	GAGCACCGTGAAGATGATGA
KDR	human	GGTGACCTGGATGAGAAGGA	TTCAGCTTGTGGATGTGCTC
LDLR	human	ACCACGGTGGAGATAGTGACA	CTTAAGCCGCCAGTTCTTCCA
NRP1	human	CGCTACCAGAAGCCAGAGGA	CATCCACAGCAATCCCACCAA
OLR1	human	GCGACTCTAGGGGTCCTTTG	GTGAGTTAGGTTTGCTTGCTCT
RPL19	human	CGCACATGGGCATAGGTAAG	CCATGAGAATCCGCTTGTTT
SCARB1	human	GGTCCCTGTCATCTGCCAAA	CTCAGGACCCTACAGTTTTGCT
SLC2A1	human	CTTCACTGTCGTGTCGCTGT	TGAAGAGTTCAGCCACGATG
SLC2A2	human	GGAGTTGGCGCTGTAAACAT	AAACTCAGCCACCATGAACC
SLC2A3	human	TCGCATCATTGCACTCTAGC	AAATGGGACCCTGCCTTACT
SLC2A4	human	CTTCGAGACAGCAGGGGTAG	AGGAGCAGAGCCACAGTCAT
SLC2A5	human	GCTTGATCTTCCCGTTCATC	CCTTTTCCGGGTACACTTCA
SLC2A6	human	GGGGCTGTACATCCACTTTG	CCATGATGAAGAGCATGGTG
SLC2A7	human	CTGGCGTCGTCAACATAGTG	ATGATGCCGAGGTAGGACAG
SLC2A8	human	CAACGCCGTCATGTTCTATG	CCATGACCACACCTGACAAG
SLC2A9	human	GCATCCCGTTCATCTTGACT	GGTCTCAGGCAGCACAAAAT
SLC2A10	human	CCCTGTGGAGATACGAGGAA	GACTGGCCTTTTGTTTCAGG
SLC2A11	human	GAAGCGAAGATCCAGTACGC	AAGCTGAGGATGAAGGCAAA
SLC2A12	human	TGCTGGATTAAGCCACACTG	GGAAAGATCTCGCTGAGGAC
SLC2A13	human	AGATGCGTGGTAACCAGACC	CTGCAGAATGGTTGCACTGT
SLC2A14	human	ATGAAGCCGCACACCCTCGCAG	GGGCATTTGCCTTGTCCGTCAAAGTTTTATT
Abca1	mouse	TGGCAAGGTTGGTGAATGGG	AGAGCTTTCGTTTGTTGCCG
Dhcr7	mouse	GCCCTACCTCTACACACTGC	ACAGGTCCTTCTGATGGTTGG
Flt1	mouse	GGAGGAGTACAACACCACGG	GGTGACCTGGATGAGAAGGA
Gyg	mouse	CACCACCAACGTCTTACCCC	CTACAGAAGCCACAAGAGAAAGC
Gys1	mouse	TGCCCATGTCTTCACTACCG	CTTCACATTCAGCCCGTTGG
Hk1	mouse	CTACTGGCATATTACTTCACCGAGC	AATTTCATCAGAGAGCCGCATGG
Hk2	mouse	GCTAGGAGCTACCACACACC	CTGTCACCCTTACTCGGAGC
Hmgcr	mouse	AATTGAACTCCCCATCGAGCC	GTAAGCTGGGATATGCTTGGC
Hmgcs1	mouse	GGAGACCACAGTTCTCTGTCC	TTCAAAGGAAGTGACCCAGGC
Ldlr	mouse	GCTCCATAGGCTATCTGCTCTTCA	CTGCGGTCCAGGGTCATC
Npc1	mouse	GTTCCGAAACCGCCCGC	CTCCATACCAAACACAGGATTGC
Nrp1	mouse	GGAGCTACTGGGCTGTGAAG	CCTCCTGTGAGCTGGAAGTC
Pcsk9	mouse	GCGAATTATCCCAGCATGGC	GAGATACACCTCCACCTGGC
Pcx	mouse	GGGCGGAGCTAACATCTACC	ACATTTGGGGAGGCAACAGG
Pfkfb3	mouse	AGCTACGAAGATGCCGTTGG	CATCACAATCACGGTTGGGG
Pygm	mouse	CCTGTGCACTTCTATGGCCG	TGTCATAGGGCATAGCCAGC
Rpl19	mouse	GGTGACCTGGATGAGAAGGA	TTCAGCTTGTGGATGTGCTC
Scarb1	mouse	AAAAGGGCTCCCAGGATAAGG	CCAAGCTATCAGGTTTGGGGG
Slc2a1	mouse	ATTGTGGCCGAGCTGTTC	GAGCACCGTGAAGATGATGA
Slc2a4	mouse	GATTCTGCTGCCCTTCTGTC	ATTGGACGCTCTCTCTCCAA
Soat1	mouse	GACCTGCTCTGGTTTTTCTCG	AGTAACTCAGGCAGATGGCG
Ugp2	mouse	GGAGAGAGACCTTCCTTACAACG	TGACCTCTTGGAACTGAGAAGC
Vegfb	mouse	TCTGAGCATGGAACTCATGG	TCTGCATTCACATTGGCTGT

Table S3. Selected genes from HBMEC microarray shown in Fig EV4

Target gene	Target protein
Pentose Phosphate Pathway	
ALDOA	aldolase A, fructose-bisphosphate
ALDOB	aldolase B, fructose-bisphosphate
ALDOC	aldolase C, fructose-bisphosphate
DERA	deoxyribose-phosphate aldolase (putative)
FBP1	fructose-1,6-bisphosphatase 1
FBP2	fructose-1,6-bisphosphatase 2
G6PD	glucose-6-phosphate dehydrogenase
GPI	glucose-6-phosphate isomerase
H6PD	hexose-6-phosphate dehydrogenase (glucose 1-dehydrogenase)
PFKL	phosphofructokinase
PFKM	phosphofructokinase
PFKP	phosphofructokinase
PGD	phosphogluconate dehydrogenase
PGLS	6-phosphogluconolactonase
PGM1	phosphoglucomutase 1
PGM3	phosphoglucomutase 3
PRPS1	phosphoribosyl pyrophosphate synthetase 1
PRPS1L1	phosphoribosyl pyrophosphate synthetase 1-like 1
PRPS2	phosphoribosyl pyrophosphate synthetase 2
RBKS	ribokinase
RPE	ribulose-5-phosphate-3-epimerase
RPIA	ribose 5-phosphate isomerase A
TALDO1	transaldolase 1
TKT	transketolase
TKTL1	transketolase-like 1
TKTL2	Transketolase-like 2
FAO/FA degradation	
ACAT2	acetyl-CoA acetyltransferase 2
ACAT1	acetyl-CoA acetyltransferase 1
ACAA1	acetyl-CoA acyltransferase 1
ACAA2	acetyl-CoA acyltransferase 2
ACOX3	acyl-CoA oxidase 3
ACOX1	acyl-CoA oxidase 1
ACOX2	acyl-CoA oxidase 2
ACADS	acyl-CoA dehydrogenase short chain
ACADM	acyl-CoA dehydrogenase medium chain
ACADL	acyl-CoA dehydrogenase long chain
ACADSB	acyl-CoA dehydrogenase short/branched chain
ACADVL	acyl-CoA dehydrogenase very long chain
ACSL1	acyl-CoA synthetase long chain family member 1
ACSL3	acyl-CoA synthetase long chain family member 3
ACSL4	acyl-CoA synthetase long chain family member 4
ACSL5	acyl-CoA synthetase long chain family member 5
ACSL6	acyl-CoA synthetase long chain family member 6
ADH1A	alcohol dehydrogenase 1A (class I)
ADH1B	alcohol dehydrogenase 1B (class I)
ADH1C	alcohol dehydrogenase 1C (class I)

ADH7	alcohol dehydrogenase 7 (class IV)
ADH4	alcohol dehydrogenase 4 (class II)
ADH5	alcohol dehydrogenase 5 (class III)
ADH6	alcohol dehydrogenase 6 (class V)
ALDH3A2	aldehyde dehydrogenase 3 family member A2
ALDH1B1	aldehyde dehydrogenase 1 family member B1
ALDH7A1	aldehyde dehydrogenase 7 family member A1
ALDH9A1	aldehyde dehydrogenase 9 family member A1
AUH	AU RNA binding protein/enoyl-coenzyme A hydratase
BDH2	3-hydroxybutyrate dehydrogenase, type 2
CPT1A	carnitine palmitoyltransferase 1A
CPT1B	carnitine palmitoyltransferase 1B
CPT1C	carnitine palmitoyltransferase 1C
CPT2	carnitine palmitoyltransferase 2
CYP4A11	cytochrome P450 family 4 subfamily A member 11
CYP4A22	cytochrome P450 family 4 subfamily A member 22
ECHS1	enoyl-CoA hydratase, short chain 1
ECI1	enoyl-CoA delta isomerase 1
ECI2	enoyl-CoA delta isomerase 2
EHHADH	enoyl-CoA hydratase and 3-hydroxyacyl CoA dehydrogenase
GCDH	glutaryl-CoA dehydrogenase
HADH	hydroxyacyl-CoA dehydrogenase
HADHA	hydroxyacyl-CoA dehydrogenase trifunctional multienzyme complex subunit alpha
HADHB	hydroxyacyl-CoA dehydrogenase trifunctional multienzyme complex subunit beta
Glycogen synthesis	
GBE1	glycogen branching enzyme
GSK3	glycogen synthase kinase 3
GYG1	glycogenin
GYG2	glycogenin
GYS1	glycogen synthase 1
GYS2	glycogen synthase 2
PHKA1	phosphorylase kinase 1 subunit alpha
PHKG1	phosphorylase kinase 1 subunit gamma
PHKB	phosphorylase kinase 1 subunit beta
PP1	phosphoprotein phosphatase 1
PTG (PPP1R3A)	protein targeting glycogen
Glycogenolysis	
PGM1	Phosphoglutomutase 1
PGM2	Phosphoglutomutase 2
PGM3	Phosphoglutomutase 3
PGM5	Phosphoglutomutase 5
PYGL	glycogen phosphorylase
PYGM	glycogen phosphorylase
PYGB	glycogen phosphorylase
Glycolysis	
ALDOA	aldolase A, fructose-bisphosphate
ALDOB	aldolase B, fructose-bisphosphate
ALDOC	aldolase C, fructose-bisphosphate
BPGM	bisphosphoglycerate mutase
ENO1	enolase 1
FBP1	fructose-1,6-bisphosphatase 1

FBP2	fructose-1,6-bisphosphatase 2
GAPDH	glyceraldehyde-3-phosphate dehydrogenase
GPI	glucose-6-phosphate isomerase
HK1	Hexokinase 1
HK2	Hexokinase 2
LDHA	lactate dehydrogenase A
LDHB	lactate dehydrogenase B
LDHD	lactate dehydrogenase D
PDK1	pyruvate dehydrogenase kinase 1
PFKFB3	6-phosphofructo-2-kinase/fructose-2,6-biphosphatase 3
PFKM	phosphofructokinase
PGAM1	phosphoglycerate mutase 1
PGAM2	phosphoglycerate mutase 2
PGAM5	phosphoglycerate mutase 5
PGK1	phosphoglycerate kinase 1
PKM1	pyruvate kinase
PKM2	pyruvate kinase
SLC16A1	MCT1 (monocarboxylate transporter)
SLC16A7	MCT2 (monocarboxylate transporter)
SLC16A3	MCT3 (monocarboxylate transporter)
SLC16A4	MCT4/MCT5
SLC16A5	MCT5/MCT6
SLC16A6	MCT6/MCT7
TIGAR	TP53 induced glycolysis regulatory phosphatase
FA synthesis	
ACACA	acetyl-CoA carboxylase alpha
ACACB	acetyl-CoA carboxylase beta
ACSF3	acyl-CoA synthetase family member 3
MCAT	malonyl-CoA-acyl carrier protein transacylase
FASN	fatty acid synthase
OXSM	3-oxoacyl-ACP synthase, mitochondrial
CBR4	carbonyl reductase 4
HSD17B8	hydroxysteroid 17-beta dehydrogenase 8
HTD2	hydroxyacyl-thioester dehydratase type 2
MECR	mitochondrial trans-2-enoyl-CoA reductase
OLAH	oleoyl-ACP hydrolase

Appendix Figure S1 (Related to Fig 2)

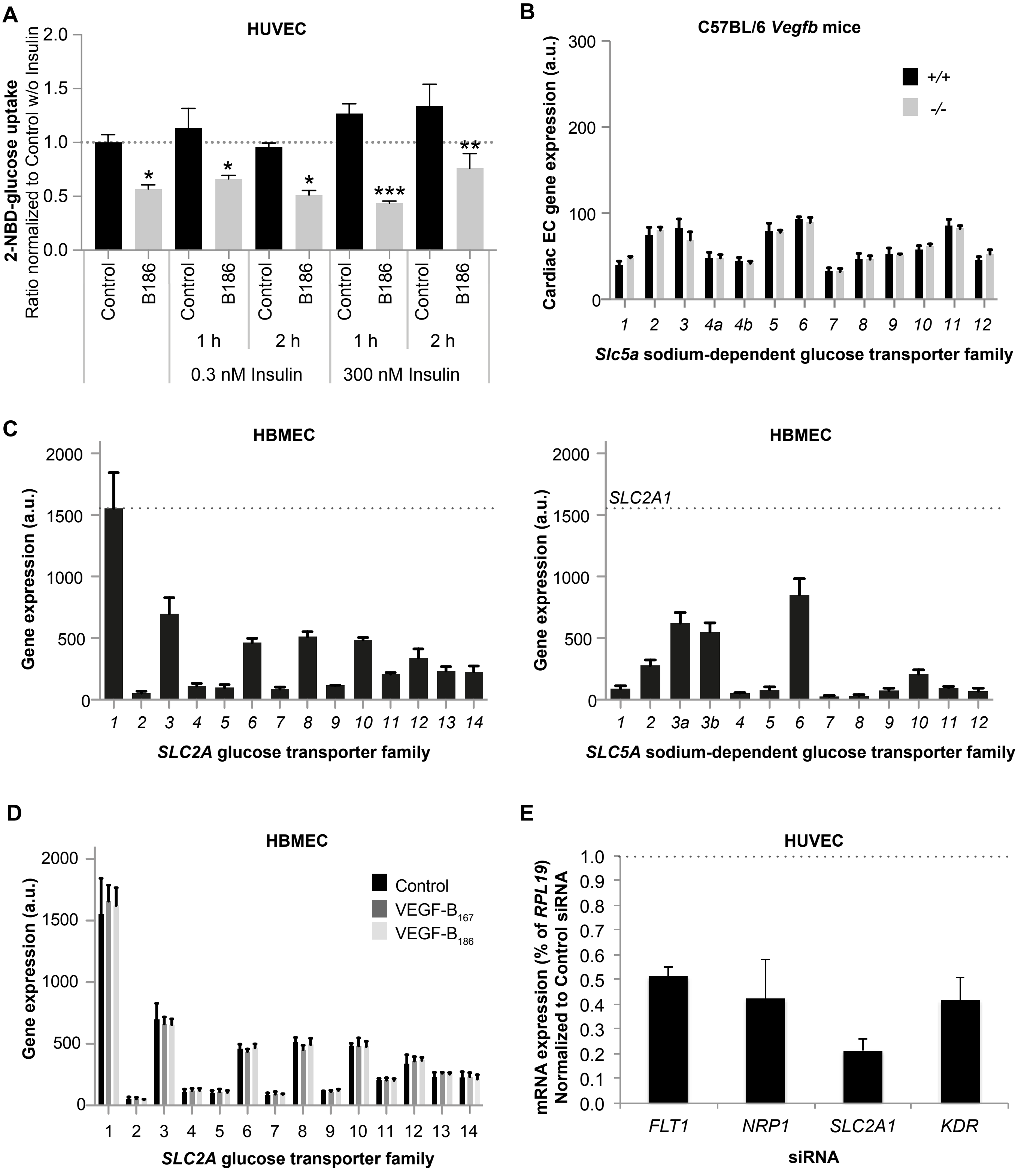

Figure S1. Characterization of endothelial glucose uptake (Related to Fig 2)
Figure legend on next page.

Figure S1. Characterization of endothelial glucose uptake (Related to Fig 2)

A 2-NBD-glucose uptake in primary human umbilical vein endothelial cells (HUVEC) treated with VEGF-B_{186} (B186) in physiological (0.3 nM) and high (300 nM) insulin concentrations. Data presented as mean ± SEM from a representative experiment performed in triplicates. Statistical evaluation using one-way ANOVA and Sidak´s multiple comparisons test, *p*-value: ** <0.01, *** <0.001 (compared to respective control).

B Relative mRNA expression of the *Slc5A* sodium-glucose transporter (SGLT) family members (*1-12*) in cardiac ECs derived from *Vegfb*$^{+/+}$ and *Vegfb*$^{-/-}$ mice. Data represent normalized PLIER values from microarray analysis.

C Relative mRNA expression of the *SLC2A1-SLC2A14* (GLUT1-GLUT14) (left panel) and *SLC5A1-SLC5A12* (SGLT1-SGLT12) (right panel) glucose transporter family members in primary human brain microvascular endothelial cells (HBMEC). The dotted line represents the *SLC2A1* (GLUT1) level for comparison. Data represent normalized PLIER values from microarray analysis.

D Effect on expression of *SLC2A* (GLUT) glucose transporter family members in primary human brain microvascular endothelial cells (HBMEC) treated for 6 h with VEGF-B_{167} or VEGF-B_{186}. Data represent normalized PLIER values from microarray analysis.

E mRNA expression levels of *FLT1* (VEGFR1), *NRP1*, *SLC2A1* (GLUT1) and *KDR* (VEGFR2) following siRNA targeting in primary human umbilical vein endothelial cells (HUVEC). Data presented as mean ± StDev relative to *RPL19* expression.

Appendix Figure S2 (Related to Fig 3)

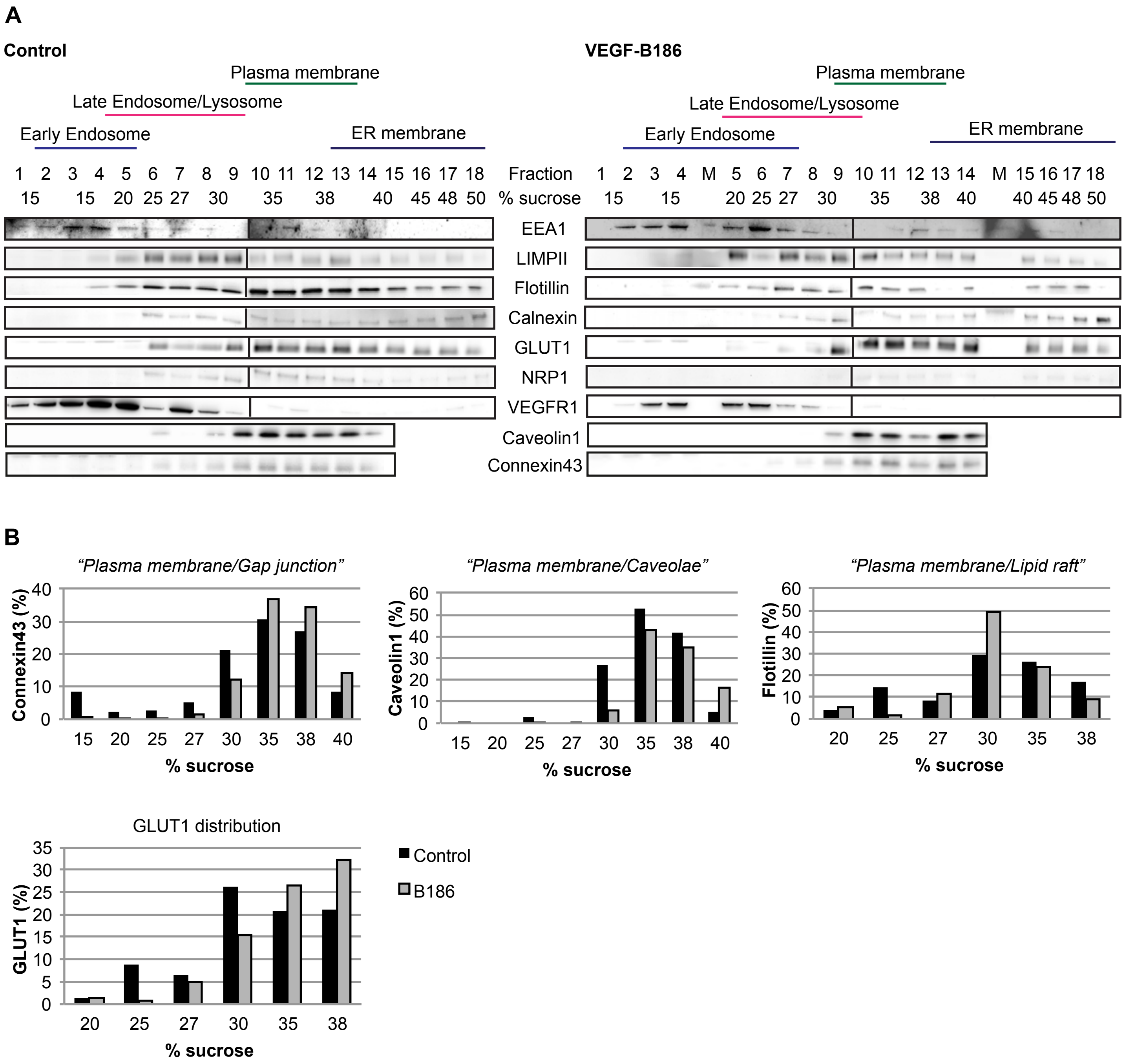

Figure S2. VEGF-B signaling changes microdomain localization of GLUT1 in the plasma membrane (Related to Fig 3)

Figure legend on next page.

Figure S2. VEGF-B signaling changes microdomain localization of GLUT1 in the plasma membrane (Related to Fig 3)

A Representative Western blots of cellular membrane fractions derived by sucrose gradient ultracentrifugation of primary human umbilical vein endothelial cell (HUVEC) lysates in response to 2 h treatment with VEGF-B_{186}. EEA1 (early endosome marker), LIMPII (late endosome/lysosome marker), Flotillin (plasma membrane/lipid raft marker), Calnexin (ER marker), GLUT1, NRP1, VEGFR1, Caveolin1 (plasma membrane/caveolae marker) and Connexin43 (plasma membrane/gap junction marker) antibodies are shown.

B Protein distribution pattern in the different sucrose fractions representing different plasma membrane subdomains. Data represent % protein relative to total protein of interest measured from a representative experiment.

Figure S8. VEGF-B stimulation reduces dil-LDL binding and uptake. Related to Figure 6.

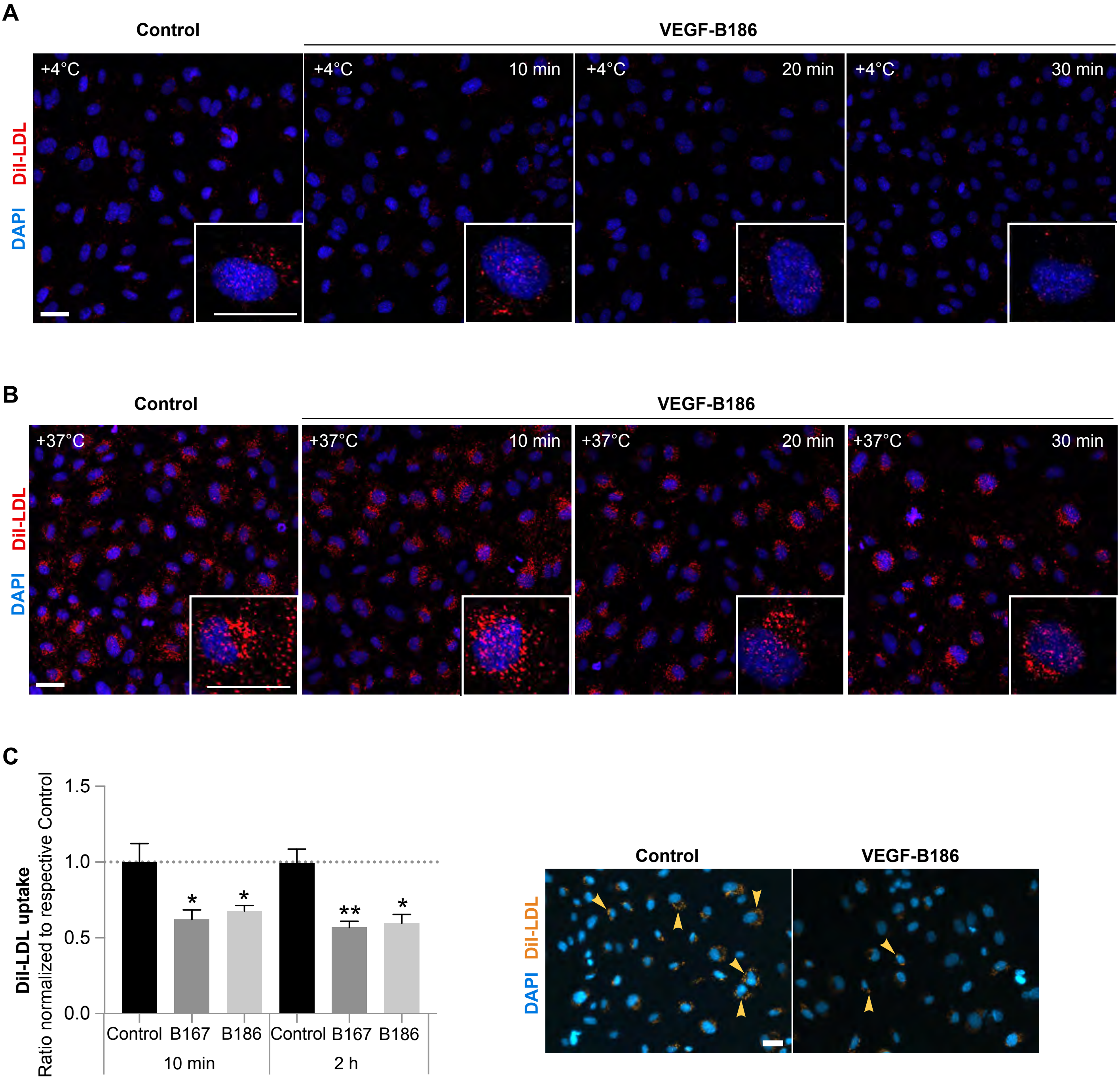

Figure S3. VEGF-B stimulation reduces DiI-LDL binding and uptake (Related to Fig 6)

A-B Representative images from DiI-LDL binding (A) and uptake (B) assays performed in primary human umbilical vein endothelial cells (HUVEC). Detailed view in higher magnification in insets, also shown in Fig 6. DiI-LDL cell surface binding evaluated at 4 °C (A) and DiI-LDL uptake (evaluated at 37 °C) in response to 10 min, 20 min and 30 min of VEGF-B_{186} stimulation. Scale bars, 10 µm.

C Quantification of DiI-LDL uptake in HUVECs after 10 min and 2 h of VEGF-B stimulation. Representative images after 2 h of VEGF-B_{186} stimulation (right panels). Arrow heads point to cells accumulating LDL. Data presented as mean ± SEM from a representative experiment performed in triplicates. Statistical evaluation using one-way ANOVA and Dunnett´s multiple comparisons test, *p*-value: * <0.05, ** <0.01 (compared to the 10 min control). Scale bar, 20 µm.

Appendix Figure S4 (Related to Fig 6)

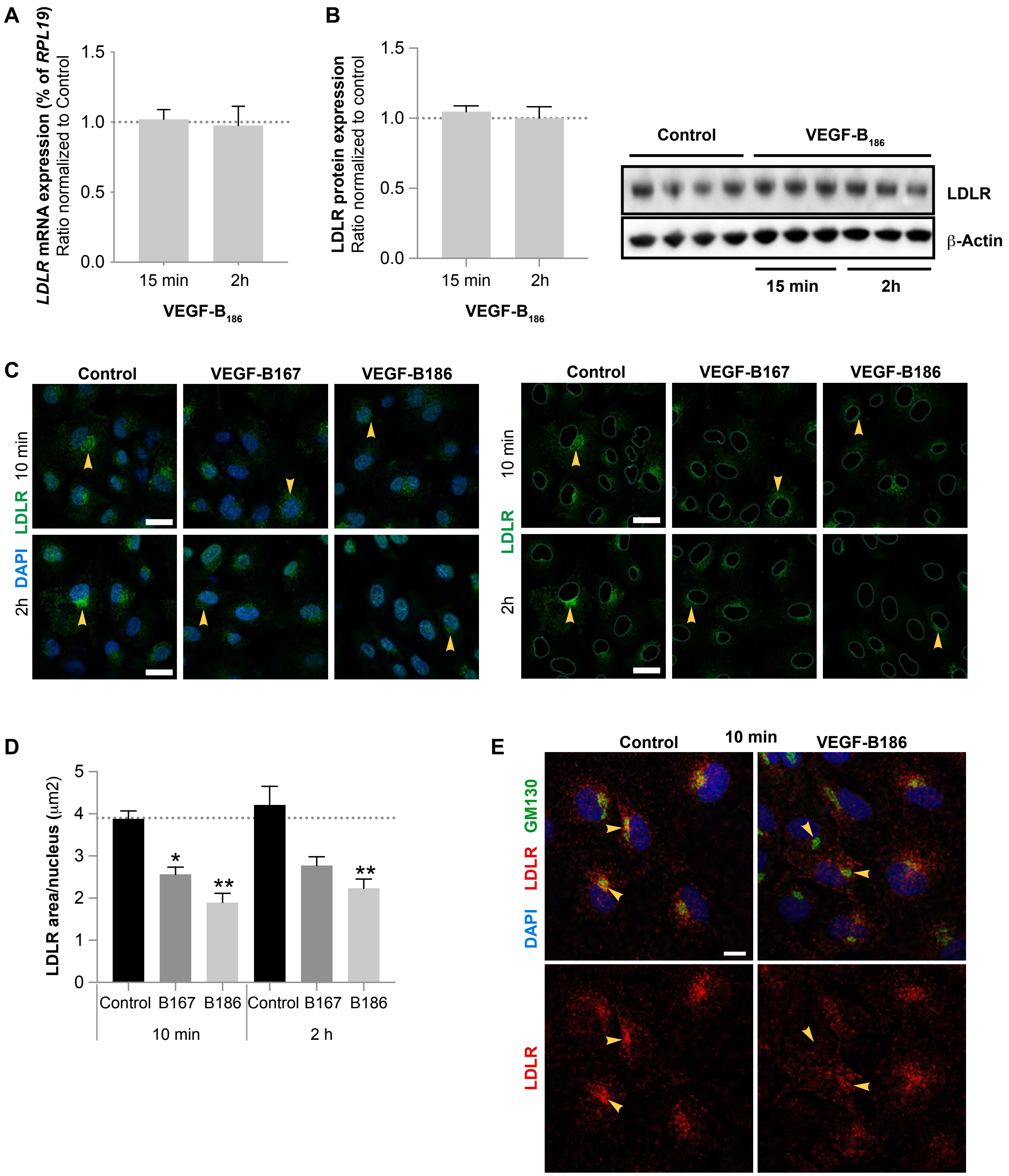

Figure S4. Decreased perinuclear LDLR distribution in response to VEGF-B signaling (Related to Fig 6)
Figure legend on next page.

Figure S4. Decreased perinuclear LDLR distribution in response to VEGF-B signaling (Related to Fig 6)

A *LDLR* mRNA expression in response to 15 min and 2 h VEGF-B_{186} treatment in primary human umbilical vein endothelial cells (HUVEC). Data presented as mean ± StDev of 3 independent experiments relative to *RPL19* expression. Statistical evaluation using *t*-test revealed no significant changes.

B LDLR protein expression in response to 15 min and 2 h VEGF-B_{186} treatment in HUVECs. Data presented as mean ± StDev of 3-4 independent experiments relative to β-Actin expression. Statistical evaluation using *t*-test revealed no significant changes. Western blots shown in right panel.

C Immunofluorescence labeling of LDLR (green) and nuclei (blue) in HUVECs after 10 min and 2 h of VEGF-B_{167} or VEGF-B_{186} stimulation. In the right set of panels, nuclei (DAPI+) have been omitted from the images in the left set of panels for better visualization of the LDLR. Representative images are shown. Arrow heads point to a less dense perinuclear LDLR distribution in VEGF-B stimulated cells. Scale bar, 10µm.

D Quantification of LDLR protein cluster area normalized to cell number in HUVECs after 10 min or 2 h of VEGF-B_{167} or VEGF-B_{186} stimulation. Data presented as mean ± SEM from three independent experiments. Statistical evaluation using one-way ANOVA and Dunnett´s multiple comparisons test, *p*-value: * <0.05, ** <0.01 (compared to the 10 min control).

E Immunofluorescence labeling of LDLR (red), early Golgi marker (GM130, green) and nuclei (blue) in HUVECs after 10 min of VEGF-B_{186} stimulation. Representative images are shown. Arrow heads points to less accumulation of LDLR around the Golgi compartment after VEGF-B_{186} stimulation. Scale bar, 10µm.

Appendix Figure S5 (Related to Fig 6)

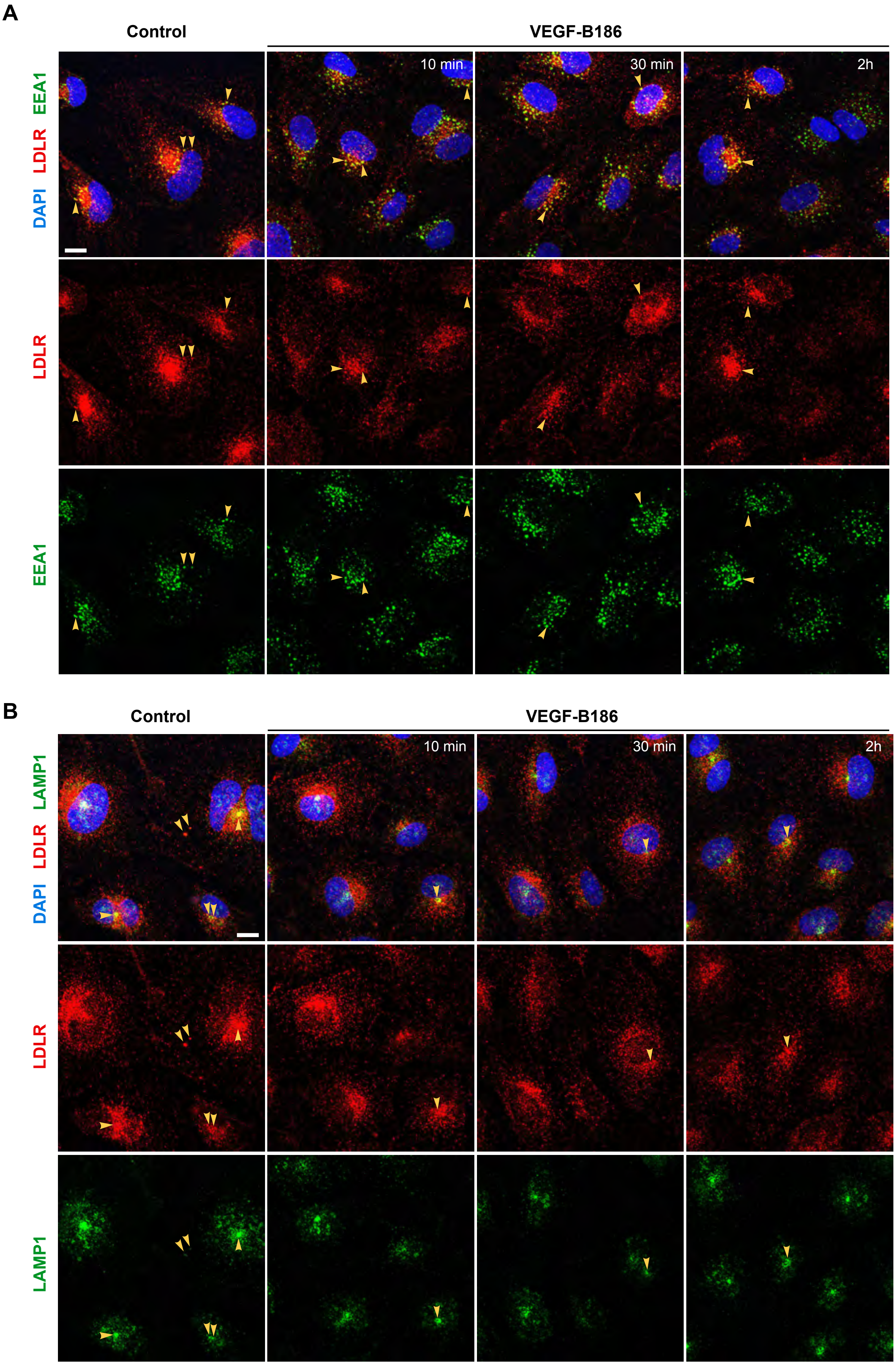

Figure S5. LDLR distribution in response to VEGF-B signaling (Related to Fig 6)
Figure legend on next page.

Figure S5. LDLR distribution in response to VEGF-B signaling (Related to Fig 6)

A Immunofluorescence labeling of LDLR (red), Early endosome marker (EEA1, green) and nuclei (blue) in primary human umbilical vein endothelial cells (HUVEC) after 10 min, 30 min and 2 h of VEGF-B_{186} stimulation. Representative images are shown. Arrow heads point to co-localization of LDLR+ areas and EEA1+ endosomes. Scale bar, 10μm.

B Immunofluorescence labeling of LDLR (red), lysosome marker LAMP1 (green) and nuclei (blue) in HUVECs after 10 min, 30 min and 2 h of VEGF-B_{186} stimulation. Representative images are shown. Arrow heads point to co-localization of LDLR+ areas and LAMP1+ lysosomes. Scale bar, 10μm.

Appendix Figure S6 (Related to Fig 7)

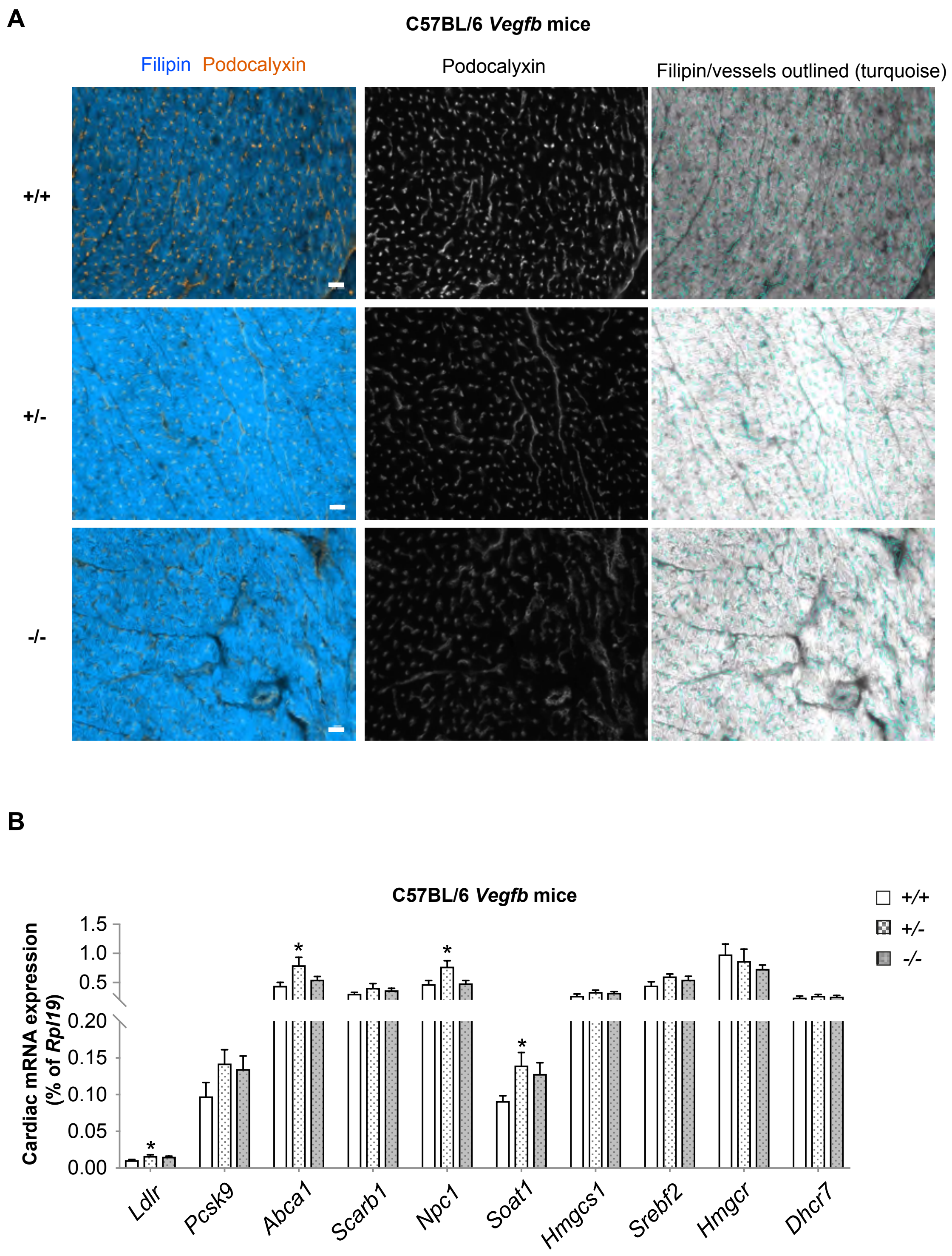

Figure S6. VEGF-B influences vessel-associated cholesterol (Related to Fig 7)
Figure legend on next page.

Figure S6. VEGF-B influences vessel-associated cholesterol (Related to Fig 7)

A Representative images of filipin (non-esterified cholesterol) and podocalyxin (endothelial cell marker) co-staining in cryo-sections of murine hearts derived from 15 w old male C57BL/6 *Vegfb*$^{+/+}$ (n=8), *Vegfb*$^{+/-}$ (n=5) and *Vegfb*$^{-/-}$ (n=7) mice. Scale bar, 50µm. The podocalyxin-positive areas selected and outlined in turquoise in the filipin-stained images to the right were used for quantification of filipin signal shown in Fig 7A.

B mRNA expression analysis of genes involved in cholesterol metabolism in cardiac tissue derived from *Vegfb*$^{+/+}$ (n=6), *Vegfb*$^{+/-}$ (n=6) and *Vegfb*$^{-/-}$ (n=6) mice. Data presented as mean ± StDev relative to *Rpl19* expression. Statistical evaluation using one-way ANOVA and Fisher´s LSD test, *p*-value: * <0.05 (compared to *Vegfb*$^{+/+}$).
