# Supplementary material for: VEGF‐B signaling impairs endothelial glucose transcytosis by decreasing membrane cholesterol content
Source: EMBO Rep. 2020 May 24;21(7):e49343. doi: 10.15252/embr.201949343 (PMC7332976; doi:10.15252/embr.201949343)
Supplement: Supplementary file 2 — Expanded View Figures PDF [file EMBR-21-e49343-s002.pdf]

Expanded View Figures

Figure EV1. VEGF-B regulates fatty acid and glucose uptake in endothelial cells of different origin.

A Bodipy-C_{12}-FA uptake in murine pancreatic endothelial cells (MS1, panels to the left) and primary human umbilical vein endothelial cells (HUVEC, panels to the right) treated for 2 h with VEGF-B_{167} (B167) or VEGF-B_{186} (B186). Data presented as mean ± SEM from representative experiments performed in triplicates. Statistical evaluation using one-way ANOVA and Dunnett's multiple comparison test, *P*-value: *< 0.05, **< 0.01 (compared to untreated control). Scale bar, 20 μm.

B 2-NBD-glucose uptake (panels to the left) and Bodipy-C_{12}-FA uptake (panels to the right) in primary human brain microvascular endothelial cells (HBMEC) treated for 2 h with VEGF-B_{167} (B167) or VEGF-B_{186} (B186). Data presented as mean ± SEM from representative experiments performed in triplicates. Statistical evaluation using one-way ANOVA and Dunnett's multiple comparison test, *P*-value: *< 0.05, **< 0.01 (compared to untreated control). Scale bar, 20 μm.

C 2-NBD-glucose uptake (panels to the left) and Bodipy-C_{12}-FA uptake (panels to the right) in primary human cardiac microvascular endothelial cells (HCMEC) treated for 2 h with VEGF-B_{167} (B167) or VEGF-B_{186} (B186). Data presented as mean ± SEM from representative experiments performed in triplicates. Statistical evaluation using one-way ANOVA and Dunnett's multiple comparison test, *P*-value: *< 0.05, **< 0.01 (compared to untreated control). Scale bar, 20 μm.

D 2-NBD-glucose uptake in murine pancreatic endothelial cells (MS1) and primary human umbilical vein endothelial cells (HUVEC) treated for 10 min and 2 h with VEGF-A_{165} (A165). Data presented as mean ± SEM from a representative experiment performed in triplicates. Statistical evaluation using one-way ANOVA and Dunnetts multiple comparison test revealed no significant differences.

E The dynamics of 2-NBD-glucose uptake in primary human umbilical vein endothelial cells (HUVEC) is stable over time up to 40 min after adding the 2-NBD-glucose tracer. Data obtained after 10, 20, 30, or 40 min of tracer incubation and presented as mean ± SEM from a representative experiment performed in triplicates.

F mRNA expression analysis of genes involved in glycogen metabolism in cardiac tissue derived from *Vegfb*$^{+/+}$ (*n* = 6), *Vegfb*$^{+/-}$ (*n* = 6), and *Vegfb*$^{-/-}$ (*n* = 6) mice. Data presented as mean ± StDev relative to *Rpl19* expression. Statistical evaluation using one-way ANOVA and Fisher's LSD test, *P*-value: *< 0.05 (compared to *Vegfb*$^{+/+}$).

© 2020 The Authors

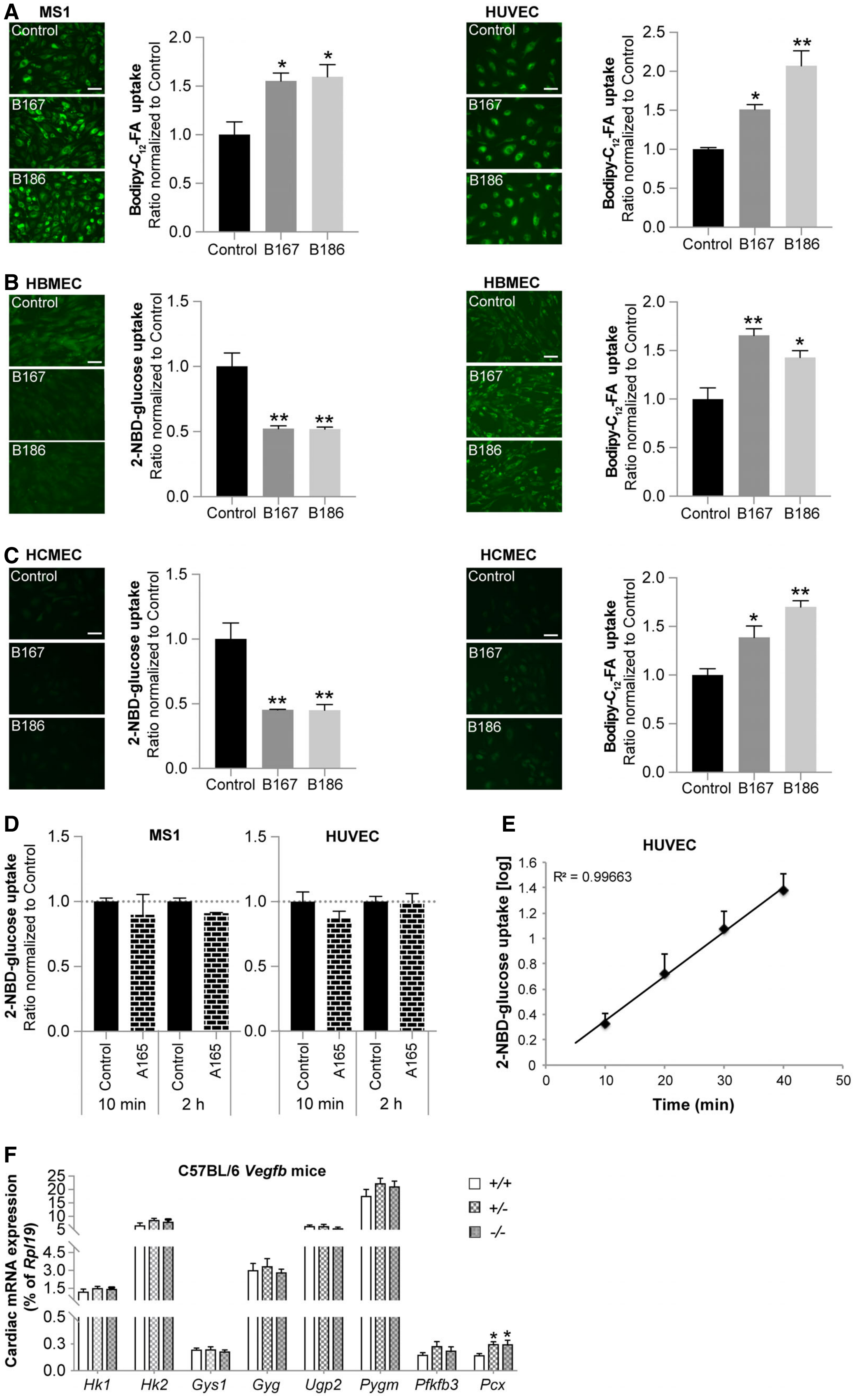

A MS1
Control
B167
B186
Bodipy-C12-FA uptake Ratio normalized to Control
Control B167 B186
HUVEC
B HBMEC
2-NBD-glucose uptake Ratio normalized to Control
HBMEC
C HCMEC
HCMEC
D
MS1
HUVEC
A165
10 min
2 h
E
HUVEC
R² = 0.99663
2-NBD-glucose uptake [log]
Time (min)
F
C57BL/6 Vegfb mice
Cardiac mRNA expression (% of Rpl19)
+/+
+/-
-/-
Hk1
Hk2
Gys1
Gyg
Ugp2
Pygm
Pfkfb3
Pcx

Figure EV1.

Figure EV2. VEGF-B regulated glucose uptake is mediated by GLUT1, NRP1, and VEGFR1.

A 2-NBD-glucose uptake in response to 2 h treatment with VEGF-B_{186} (B186) in murine pancreatic endothelial cells (MS1) exhibiting stable shRNA-mediated knockdown of *Flt1* (VEGFR1), *Nrp1*, or control shRNA (left panel). Data presented as mean ± SEM from a representative experiment performed in triplicates. Statistical evaluation using one-way ANOVA and Sidak's multiple comparison test, *P*-value: *< 0.05 (compared to respective control). ShRNA-mediated knockdown of *Flt1* (VEGFR1) and *Nrp1* expression (right panel).

B 2-NBD-glucose uptake in response to 2-h treatment with VEGF-B_{186} (B186) in the absence or presence of isopropyl β-D-1-thiogalactopyranoside (IPTG) in murine pancreatic endothelial cells (MS1) exhibiting inducible shRNA-mediated knockdown of *Slc2a1* (Glut1) (left panel). Data presented as mean ± SEM from a representative experiment performed in triplicates. Statistical evaluation using one-way ANOVA and Sidak's multiple comparison test, *P*-value: *< 0.05 (compared to respective control). ShRNA-mediated knockdown of GLUT1 protein expression (right panel).

C 2-NBD-glucose uptake in response to 2 h treatment with VEGF-B_{186} (B186) in the absence or presence of 500 nM phorbol 12-myristate 13-acetate (PMA) in primary human umbilical vein endothelial cells (HUVEC). Data presented as mean ± SEM from a representative experiment performed in triplicates. Statistical evaluation using one-way ANOVA and Tukey's multiple comparison test, *P*-value: ***< 0.001 (compared to DMSO control). *P*-value: $^{###}$ < 0.001 (PMA VEGF-B_{186} compared to PMA control).

D Bodipy-C_{12}-FA uptake in response to 2-h treatment with VEGF-B_{186} (B186) in the absence or presence of isopropyl β-D-1-thiogalactopyranoside (IPTG) in murine pancreatic endothelial cells (MS1) exhibiting inducible shRNA-mediated knockdown of *Slc2a1* (Glut1). Data presented as mean ± SEM from a representative experiment performed in triplicates. Statistical evaluation using one-way ANOVA and Sidak's multiple comparison test, *P*-value: ***< 0.001 (compared to respective control).

E Bodipy-C_{12}-FA uptake in response to 2-h treatment with VEGF-B_{186} (B186) in primary human umbilical vein endothelial cells (HUVEC) exhibiting siRNA-mediated knockdown of *SLC2A1* (GLUT1). Data presented as mean ± SEM from a representative experiment performed in triplicates. Statistical evaluation using one-way ANOVA and Sidak's multiple comparison test, *P*-value: *< 0.05, **< 0.01 (compared to respective control).

F Bodipy-C_{12}-FA uptake in response to 2-h treatment with VEGF-B_{186} (B186) in murine pancreatic endothelial cells (MS1) exhibiting stable shRNA-mediated knockdown of *Flt1* (VEGFR1), *Nrp1* or control shRNA (left panel). Data presented as mean ± SEM from a representative experiment performed in triplicates. Statistical evaluation using one-way ANOVA and Sidak's multiple comparison test, *P*-value: ***< 0.001 (compared to respective control).

 © 2020 The Authors

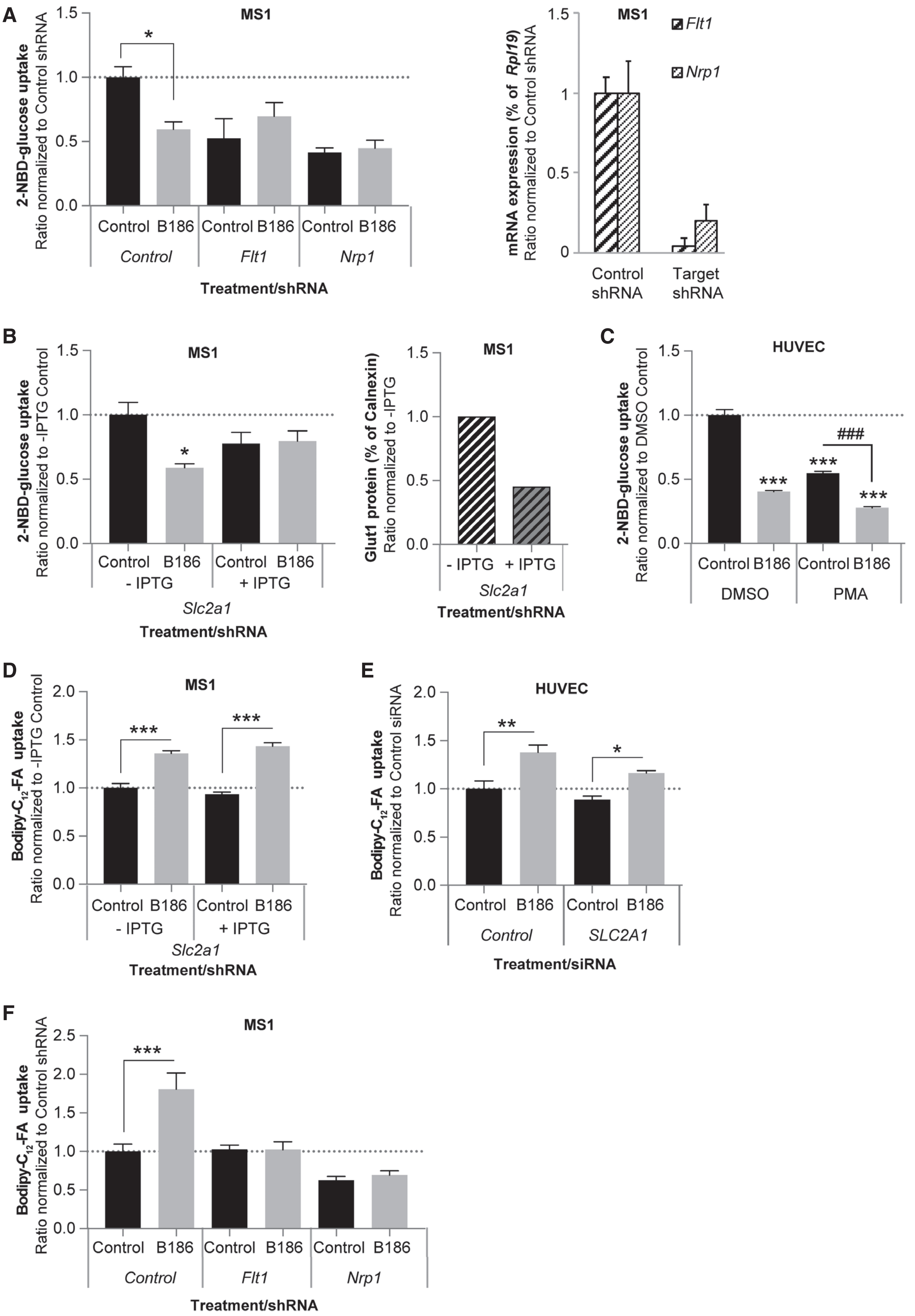
A
MS1
2-NBD-glucose uptake
Ratio normalized to Control shRNA
Control B186 Control B186 Control B186
Control Flt1 Nrp1
Treatment/shRNA
MS1
mRNA expression (% of Rpl19)
Ratio normalized to Control shRNA
Flt1
Nrp1
Control shRNA
Target shRNA
B
MS1
2-NBD-glucose uptake
Ratio normalized to -IPTG Control
Control B186 Control B186
- IPTG + IPTG
Slc2a1
Treatment/shRNA
MS1
Glut1 protein (% of Calnexin)
Ratio normalized to -IPTG
- IPTG + IPTG
Slc2a1
Treatment/shRNA
C
HUVEC
2-NBD-glucose uptake
Ratio normalized to DMSO Control
Control B186 Control B186
DMSO PMA
D
MS1
Bodipy-C12-FA uptake
Ratio normalized to -IPTG Control
Control B186 Control B186
- IPTG + IPTG
Slc2a1
Treatment/shRNA
E
HUVEC
Bodipy-C12-FA uptake
Ratio normalized to Control siRNA
Control B186 Control B186
Control SLC2A1
Treatment/siRNA
F
MS1
Bodipy-C12-FA uptake
Ratio normalized to Control shRNA
Control B186 Control B186 Control B186
Control Flt1 Nrp1
Treatment/shRNA

Figure EV2.

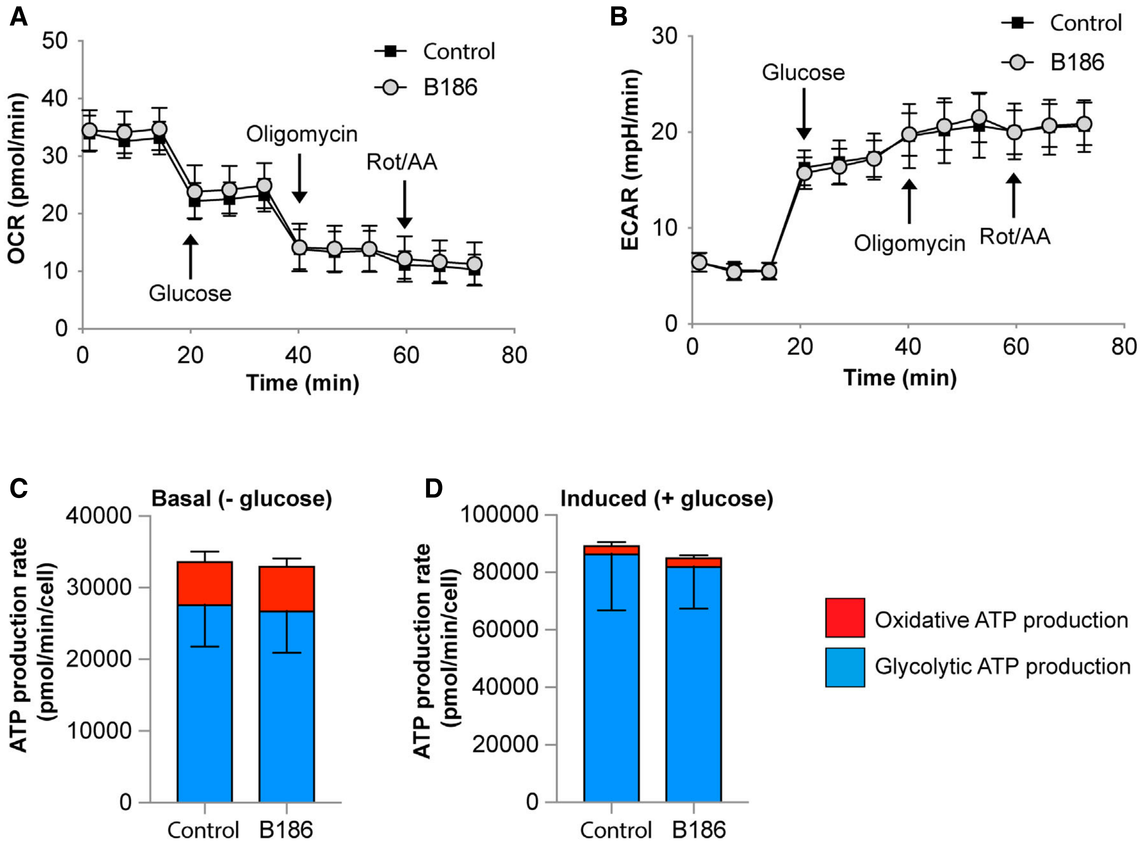

Figure EV3. VEGF-B signaling does not change endothelial energy substrate use or energy balance.

A, B Measurements of oxygen consumption rate (OCR, panel A) and extracellular acidification rate (ECAR, panel B) in primary human umbilical vein endothelial cells (HUVEC) stimulated or not with VEGF-B_{186} (B186) for 2 h using the Seahorse® live-cell metabolic assay platform. Glucose, oligomycin and rotenone + antimycin A (Rot/AA) was added at the indicated time points. Data presented as mean ± SEM from a representative experiment performed with n = 43–44 wells/condition.

C, D Comparison of oxidative (mitochondrial) versus glycolytic ATP production rate after VEGF-B stimulation during basal (glucose-free) or glucose-induced conditions. Data presented as mean ± SEM from a representative experiment performed with n = 43–44 wells/condition.

Figure EV4. VEGF-B signaling does not change expression of genes involved in endothelial cell metabolism.

RNA from primary human brain microvascular endothelial cells (HBMEC) stimulated with VEGF-B_{167}, VEGF-B_{186}, or vehicle (Control) for 6 h (n = 3 cell dishes/condition) were subjected to microarray hybridization using Affymetrix GeneChip® Human Gene 1.0 ST chips. Shown is a heat map over selected genes involved in intermediary metabolism (listed in Appendix Table S3). The three differentially expressed genes in response to VEGF-B stimulation are indicated with arrow heads.

 © 2020 The Authors

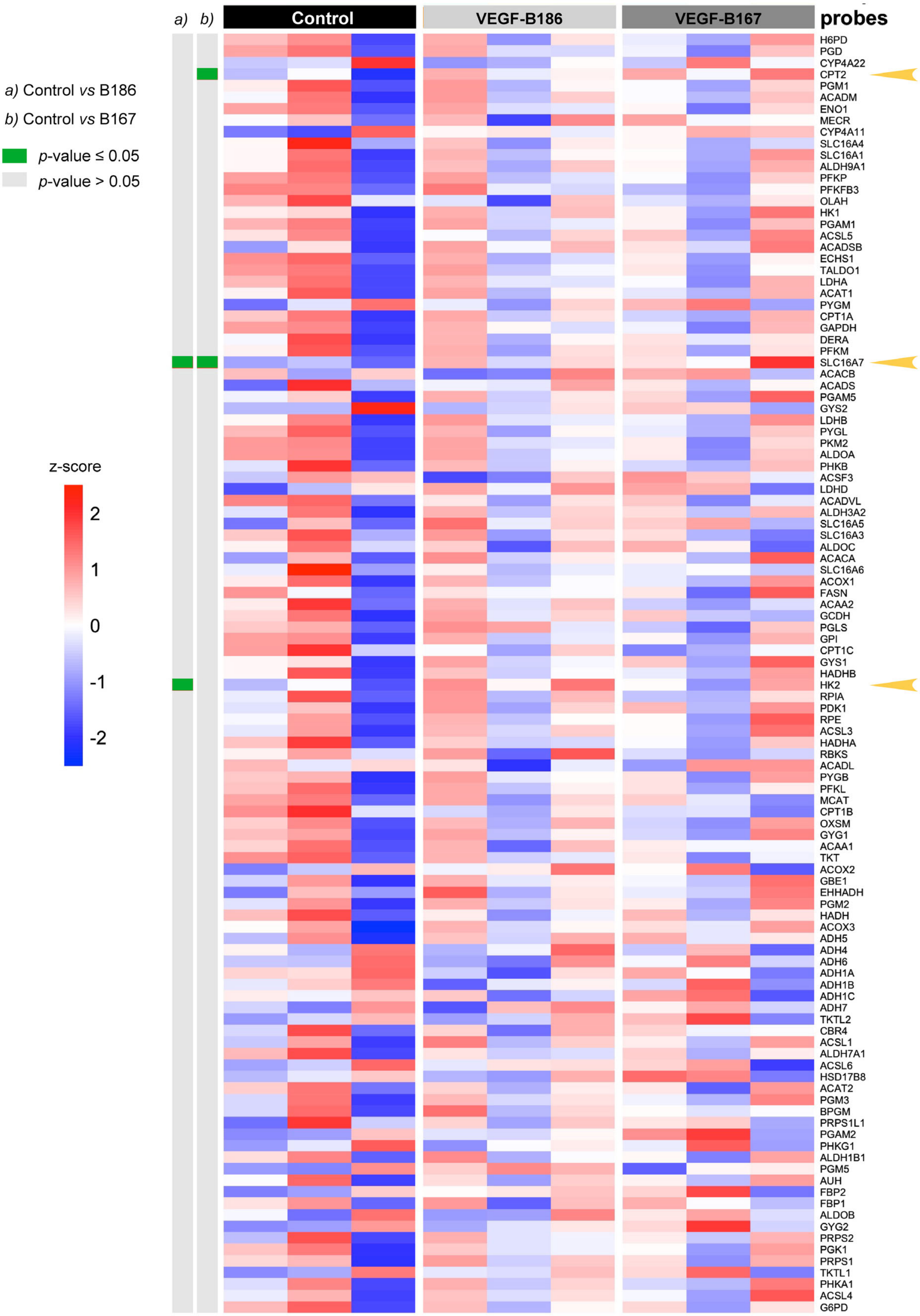
a)
b)
Control
VEGF-B186
VEGF-B167
probes
a) Control vs B186
b) Control vs B167
p-value ≤ 0.05
p-value > 0.05
z-score
2
1
0
-1
-2
H6PD
PGD
CYP4A22
CPT2
PGM1
ACADM
ENO1
MECR
CYP4A11
SLC16A4
SLC16A1
ALDH9A1
PFKP
PFKFB3
OLAH
HK1
PGAM1
ACSL5
ACADSB
ECHS1
TALDO1
LDHA
ACAT1
PYGM
CPT1A
GAPDH
DERA
PFKM
SLC16A7
ACACB
ACADS
PGAM5
GYS2
LDHB
PYGL
PKM2
ALDOA
PHKB
ACSF3
LDHD
ACADVL
ALDH3A2
SLC16A5
SLC16A3
ALDOC
ACACA
SLC16A6
ACOX1
FASN
ACAA2
GCDH
PGLS
GPI
CPT1C
GYS1
HADHB
HK2
RPIA
PDK1
RPE
ACSL3
HADHA
RBKS
ACADL
PYGB
PFKL
MCAT
CPT1B
OXSM
GYG1
ACAA1
TKT
ACOX2
GBE1
EHHADH
PGM2
HADH
ACOX3
ADH5
ADH4
ADH6
ADH1A
ADH1B
ADH1C
ADH7
TKTL2
CBR4
ACSL1
ALDH7A1
ACSL6
HSD17B8
ACAT2
PGM3
BPGM
PRPS1L1
PGAM2
PHKG1
ALDH1B1
PGM5
AUH
FBP2
FBP1
ALDOB
GYG2
PRPS2
PGK1
PRPS1
TKTL1
PHKA1
ACSL4
G6PD

Figure EV4.

Figure EV5. Fatty acid uptake by VEGF-B is cholesterol- and LDLR-independent.

A Bodipy-C_{12}-FA uptake (left panel) and corresponding measurement of cellular cholesterol content (right panel) in primary human umbilical vein endothelial cells (HUVEC) treated for 2 h with VEGF-B_{186} (B186) or 15 min with cholesterol-extracting methyl-beta-cyclodextrin (MbCD). Data presented as mean ± SEM from representative experiments performed in triplicates. Statistical evaluation using one-way ANOVA and Dunnett's multiple comparison test, *P*-value: **< 0.01, ***< 0.001 (compared to untreated control).

B Bodipy-C_{12}-FA uptake (left panel) and corresponding measurement of cellular cholesterol content (right panel) in primary human umbilical vein endothelial cells (HUVEC) treated for 2 h with VEGF-B_{186} or cholesterol-extracting methyl-beta-cyclodextrin (MbCD). Data presented as mean ± SEM from representative experiments performed in triplicates. Statistical evaluation using one-way ANOVA and Dunnett's multiple comparison test, *P*-value: *< 0.05, **< 0.01, ***< 0.001 (compared to untreated control).

C Knockdown of *LDLR* and *SCARB1* (SRB1) mRNA expression following siRNA targeting in primary human umbilical vein endothelial cells (HUVEC), normalized to levels in control siRNA-treated cells. Data presented as mean ± SEM of three independent experiments relative to *RPL19* expression. Statistical evaluation using *t*-test, *P*-value: *< 0.05, **< 0.01 (compared to respective control siRNA).

D Bodipy-C_{12}-FA uptake in response to 2-h treatment with VEGF-B_{186} (B186) in primary human umbilical vein endothelial cells (HUVEC) exhibiting siRNA-mediated knockdown of the *LDLR*. Data presented as mean ± SEM from a representative experiment performed in triplicates. Statistical evaluation using one-way ANOVA and Sidak's multiple comparison test, *P*-value: *< 0.05, **< 0.01 (compared to respective control).

E Bodipy-C_{12}-FA uptake in response to 2 h treatment with VEGF-B_{186} (B186) in primary human umbilical vein endothelial cells (HUVEC) treated with simvastatin, a *de novo* cholesterol synthesis inhibitor. Data presented as mean ± SEM from a representative experiment performed in triplicates. Statistical evaluation using one-way ANOVA and Sidak's multiple comparison test, *P*-value: *< 0.05 (compared to respective control).

© 2020 The Authors

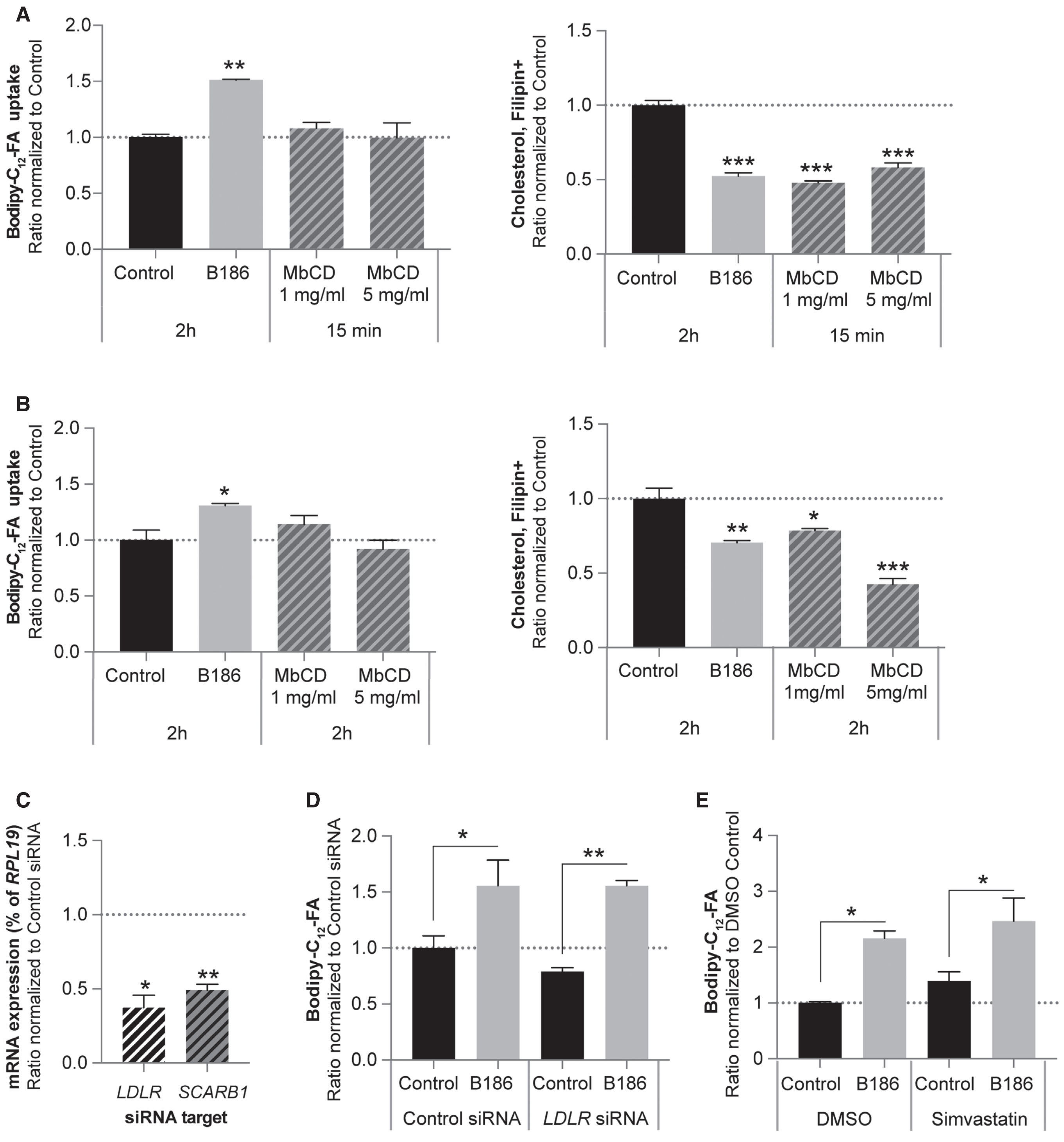
A
Bodipy-C_{12}-FA uptake
Ratio normalized to Control
2.0
1.5
1.0
0.5
0.0
**
Control
B186
MbCD 1 mg/ml
MbCD 5 mg/ml
2h
15 min
Cholesterol, Filipin+
Ratio normalized to Control
***
B
*
2h
1mg/ml
5mg/ml
**
C
mRNA expression (% of *RPL19*)
Ratio normalized to Control siRNA
LDLR
SCARB1
siRNA target
D
Bodipy-C_{12}-FA
Ratio normalized to Control siRNA
Control siRNA
LDLR siRNA
E
Bodipy-C_{12}-FA
Ratio normalized to DMSO Control
DMSO
Simvastatin

Figure EV5.
