# Supplementary figures and images for: VEGF‐B signaling impairs endothelial glucose transcytosis by decreasing membrane cholesterol content
Source: EMBO Rep. 2020 May 24;21(7):e49343. doi: 10.15252/embr.201949343 (PMC7332976; doi:10.15252/embr.201949343)

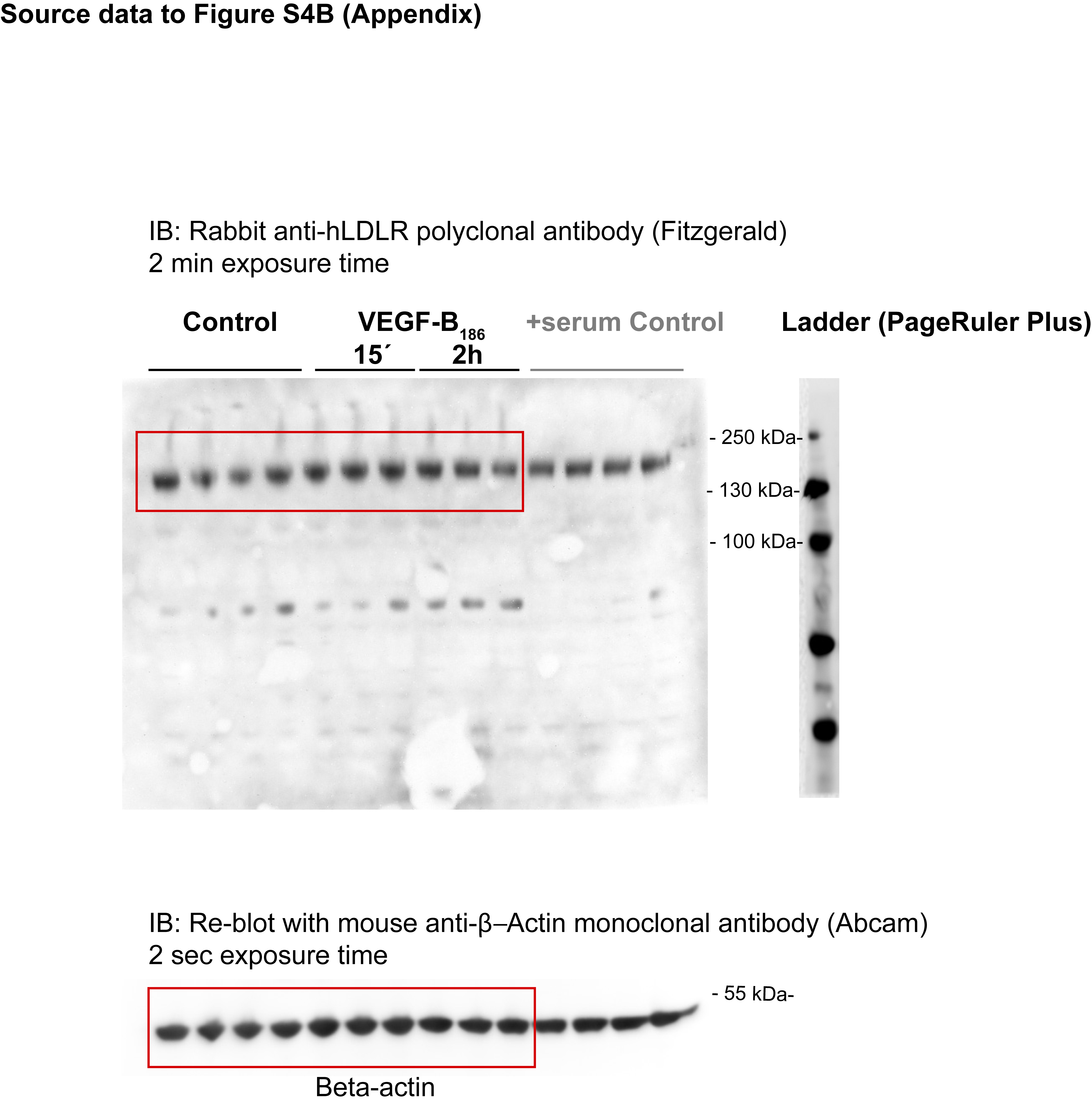

Supplement: Supplementary file 3 — Source Data for Appendix [file EMBR-21-e49343-s003.zip › embr201949343-sup-0003-SDataFigS4B.tiff]
